# Exposure Levels of Environmental Endocrine Disruptors in Mother-Newborn Pairs in China and Their Placental Transfer Characteristics

**DOI:** 10.1371/journal.pone.0062526

**Published:** 2013-05-07

**Authors:** Lu-Xi Li, Li Chen, Xiang-Zhou Meng, Bing-Heng Chen, Shang-Qin Chen, Yan Zhao, Li-Fang Zhao, Yuan Liang, Yun-Hui Zhang

**Affiliations:** 1 Key Laboratory of Public Health Safety, Ministry of Education, School of Public Health, Fudan University, Shanghai, China; 2 State Key Laboratory of Pollution Control and Resources Reuse, College of Environmental Science and Engineering, Tongji University, Shanghai, China; 3 The Second Affiliated Hospital, Wenzhou Medical College, Wenzhou, China; The Ohio State University, United States of America

## Abstract

There is a growing concern about the potential health effects of exposure to various environmental chemicals during pregnancy and infancy. The placenta is expected to be an effective barrier protecting the developing embryo against some endocrine disruptors (EDs) circulating in maternal blood. The current study was designed to assess *in utero* exposure levels of non-persistent organic pollutants (non-POPs) and persistent organic pollutants (POPs) in Chinese newborns and potential role of placenta barrier against fetal exposure to these commonly-used environmental endocrine disruptors. A total of 230 newborn-mother pairs were enrolled during 2010–2011, 201 pairs of which were recruited from Shanghai, and the other 29 pairs came from Wenzhou. Maternal blood, cord blood, and meconium specimens were collected in the subject population from Shanghai and analyzed for non-POPs, including mono-2-ethylhexyl phthalate (MEHP), octylphenol (OP) and 4-nonylphenol (4-NP). A total of 19 polybrominated diphenyl ethers (PBDEs) congeners, which belong to POPs, were detected in maternal and cord blood specimens from the other 29 pairs. Fetal-maternal ratios (F-M ratios) and regression coefficients were presented to assess potential function of placenta on barricading the mother/fetal transfer of these EDs. Concentrations of the detected non-POPs in cord blood samples were approximately 20% lower than those in maternal blood, and regression coefficients of which were all over 0.80. In contrast, PBDEs levels in cord blood samples were significantly higher than those in maternal blood. MEHP levels in meconium were much higher than those in cord blood samples, and highly correlated. Therefore, observations demonstrated that the placental barrier slightly decreased the fetal exposure to most non-POPs, while PBDEs seemed to be totally transferred across the placenta and finally reached the fetus. For *in utero* exposure assessment of Di-2-ethylhexyl phthalate (DEHP), MEHP level in meconium may be a useful biomarker.

## Introduction

Endocrine disruptors (EDs) are ubiquitous in environment matrix and human bodies. There is a growing concern about the potential health effects of exposure to various environmental chemicals during pregnancy and infancy. Mono-2-ethylhexyl phthalate (MEHP), octylphenol (OP), 4-nonylphenol (4-NP) and polybrominated diphenyl ethers (PBDEs) have been detected in physiologically relevant compartments within pregnant women and the developing fetuses, such as maternal urine, cord blood, breast milk, meconium, placenta, and amniotic fluid in several studies from Europe, the United States of America (USA), and Asia [1]–[7], showing that pregnant women and their fetuses are exposed to those chemicals.

These environmental EDs, released into the environment through production and usage, are known to mimic or antagonize hormonal activities at trace concentrations. Previous studies have indicated that exposure of pregnant women to some EDs may lead to toxic effects on mothers and to *in utero* exposure of the fetuses by blood circulating through the placenta [8]. Fetal exposure to EDs has been associated with functional developmental effects [9], and eventually may adversely affect their reproductive and hormonal systems, as well as cause cancer.

Since the developing fetuses might be more sensitive to the effects of EDs than adults [10], it is important to understand the extent and effect of prenatal exposure to these potentially toxic chemicals. The placenta is expected to be an effective barrier against fetal exposure to certain harmful proteins and also protects the developing fetuses against some EDs circulating in maternal blood that adversely affect fetal development. However, there is limited or inadequate data on the *in utero* exposure levels of Chinese newborns to these EDs so far. Most of the researches on placental transfer of these EDs were conducted by animal or *in vitro* experiments instead of human studies. Furthermore, the appropriate exposure biomarker for di-2-ethylhexyl phthalate (DEHP) *in utero* exposure assessment still needs further study. To this end, we analyzed MEHP, OP and 4-NP for non-persistent organic pollutants (non-POPs) from 201 pregnant women and their matching newborns in the blood and meconium specimens from Shanghai, and 19 PBDEs congeners for persistent organic pollutants (POPs) from 29 pregnant women and their matching newborns in blood samples from Wenzhou, China, to observe *in utero* exposure levels and whether placenta presents an efficient barrier against fetal exposure to these major EDs. Also, we still tried to find out an effective biomarker for DEHP *in utero* exposure assessment.

## Methods

### Recruitment of Participants

Two hundreds and thirty mother-newborn pairs were enrolled during 2010–2011 from the Yangtze River Delta, of which 201 pairs were from Shanghai Medical Center for Maternal and Child Health, and the other 29 pairs were from the Second Affiliated Hospital of Wenzhou Medical College, after excluding multiple-birth pregnancies and premature deliveries. And the mothers from Wenzhou were living near Taizhou, the major e-waste dismantling and recycling area in China.

All subjects signed written informed consent, and Fudan University’s Institutional Review Board specifically approved this study. The written consent was obtained from the mothers for their participation in the study, and for their infants’ participation. The mothers completed questionnaires including information about the newborn’s birth status and maternal information [e.g. medical history, menstruation, civil status, education, occupation, hobby, tobacco smoking, drinking, dietary habits (including vitamin supplementation), physical activity] after delivery within two days. All subjects were examined and investigated before the results of chemical analysis were known. Newborns’ supine length was measured using an infantometer, and birth weight measured on a digital scale, both obtained from the hospital records.

### Sample Collection

Cord blood was obtained from 230 infants immediately after delivery using a syringe. Maternal blood samples were collected after the mothers completed the questionnaire. The blood sample obtained from the mothers was about 10 mL each, and about 20 mL from every infant. After coagulation, samples were centrifuged to separate the serum which was immediately transferred to clean containers, frozen, transported to the laboratory, and then stored at −20°C.

For each infant from Shanghai Medical Center for Maternal and Child Health, meconium was collected directly from every diaper during the first 48 hours after delivery, and all of these meconium samples of every infant were pooled into a single sample. All specimens were collected with glass devices to avoid contamination by phthalates during handling and storage. Frozen samples were stored in phthalate-free containers and transferred on dry ice to the Fudan University laboratory for analysis.

### Measurement

The non-POPs, including MEHP, OP and 4-NP were analyzed in maternal blood, cord blood and meconium specimens of 201 mother-newborn pairs from Shanghai, while 19 PBDEs congeners, belonging to the POPs, were analyzed in maternal and cord blood samples of the other 29 pairs from Wenzhou.

Analyses of these EDs in serum and meconium samples were done as described previously [11], [12]. In brief, after enzymatic deconjugation of MEHP, the non-POPs in serum (1 mL) and meconium (1 g) involved solid-phase extraction, separation with an Agilent 1100 Series high-performance liquid chromatography (HPLC) system (Agilent Technologies, Santa Clara, CA), and detection by an API 2000 electrospray triple quadrupole mass spectrometer (ESI-MS/MS; Applied Biosystems, Foster City, CA).

For PBDEs, all standards (BDE-17, 28, 33, 47, 49, 50, 66, 99, 100, 118, 128, 138, 153, 154, 172, 183, 190, 196, 203, 206, 207, 208, and 209) were obtained from the Accustandards (New Haven, CT, USA). Detailed extraction and instrumental analytical procedures of PBDEs were described elsewhere [12]. We followed their procedures. Lipid content was determined gravimetrically using the whole extract. Each of the extract was cleaned by 2 mL concentrated H_2_SO_4_. Then the extract was subject to a gel permeation chromatography column (filled with Biobeads SX-3) and eluted with a mixture of dichloromethane and hexane (1∶1 in volume). The fraction from 110 to 210 mL was collected and concentrated to 100 L. The internal standards (BDE-118 and BDE-128) were added to the final extract prior to instrumental analysis. PBDEs congeners were analyzed on a Shimadzu GC/MS-QP 2010 plus equipped with a 15 mm×0.25 mm i.d.×0.1 m DB-5 (J&W Scientific, Folsom, CA, USA).

The limit of detection (LOD) of MEHP, OP and 4-NP was 1.0 ng/g for meconium and 0.2 to 1.0 µg/L for serum. As for PBDEs in serum samples, the LOD was 0.1 ng/g. Analysts at the Key Laboratory of Public Health Safety in Fudan University and State Key Laboratory of Pollution Control and Resources Reuse in Tongji University were blind to all information concerning our subjects. This study was conducted in accordance with protocols approved by Fudan University’s Ethics Committee.

### Statistical Analysis

The value of 1/2 LOD was used to estimate the value of samples below the LOD [13]. Analyses of potential differences in these EDs levels between maternal blood and cord blood, cord blood and meconium were conducted using Wilcoxon matched-pairs signed-ranks test. And multiple linear regression was used to explore their associations. For the reason of lipid-adjusted PBDE levels are non-normal distribution data, geometric mean (GM) with 95% CI was presented to characterize PBDEs levels in the descriptive analysis. A log-transformation was applied to all individual BDE congeners and their sums due to the skewed distributions, as typically occurs with environmental contaminants. The analyses were considered statistically significant when p<0.05. All statistical analyses were conducted using the SPSS 17.0 statistical package (SPSS Inc, Chicago, Illinois).

## Results

In these two subject populations, first births both represented about 80% of the newborns. The majority of the mothers had completed high school education (65% and 76%) and received 5 times prenatal care examinations (54% and 90%), are clear of occupational exposure, had regular menstruation, and lives in non-industrial area. Of the first population, nearly 6% mothers reported family members or themselves smoking or drinking alcohol at home during the entire pregnancy, while there are nearly a half of the second population. A few mothers reported to have had pregnancy complications (including pregnancy-induced hypertension, diabetes, infection, and intrahepatic cholestasis syndrome) (25% and 7%) (Table 1).

**Table 1:** Selected characteristics of subject populations.

Characteristics	Shanghai (N = 201)	Wenzhou (N = 29)
Birth weight, g^a^	2980(2400∼3480)	2870(2350∼3175)
Birth length, cm^b^	47.5±2.5	48.8±2.5
Gestational age, week^b^	38.5±1.4	40.4±2.7
Sex, male/female	103/98	10/19
Uncomplicated vaginal births, yes/no	127/74	29/0
Maternal age, years^b^	27.9±3.6	25.1±2.8
Prepregnancy BMI, </≥18.5	43/158	13/16
Prenatal care (Kessner index), adequate/inadequate	109/92	26/3
Pregnancy complications, yes/no^c^	50/151	2/27
Irregular menstruation, yes/no	38/163	2/27
Occupational exposure, yes/no	50/151	3/26
Vitamins supplement, yes/no	86/115	3/26
Maternal/paternal smoking/drinking, yes/no	12/189	14/15
Living in industrial area, yes/no	23/178	7/22
Education, </≥ high school	71/130	7/22
Income, </≥$500/month	45/156	9/20

a Data shown as median (25th∼75th percentiles).

b Data shown as mean ± standard deviation.

c Pregnancy complications include pregnancy-induced hypertension, diabetes, infection, and intrahepatic cholestasis syndrome.

**Figure 1:**
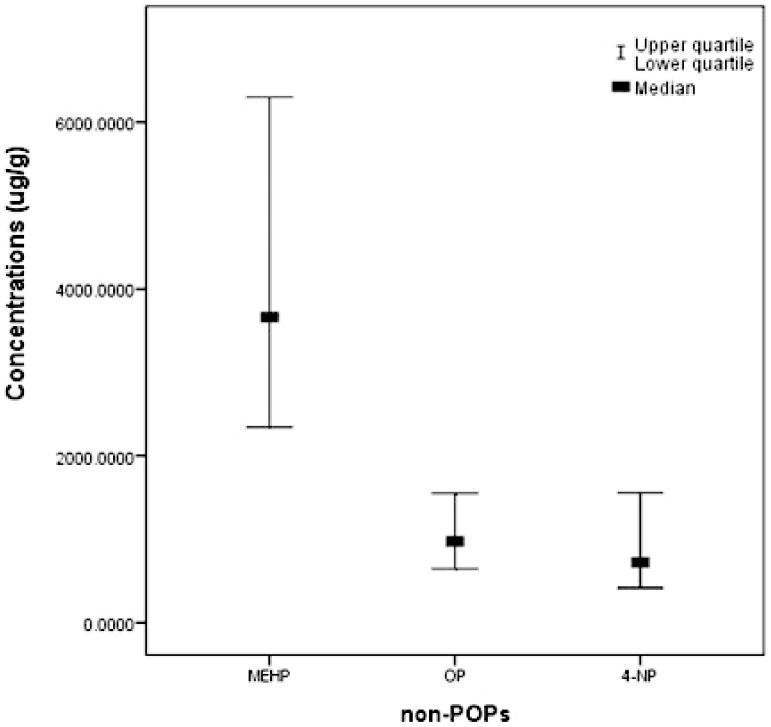
Non-POPs levels in meconium specimens. The concentrations of MEHP, OP and 4-NP in meconium specimens.

Near 65% biological samples had quantifiable levels of these non-POPs. The non-POPs levels in maternal and cord blood are reported as minimum, percentiles and maximum in Table 2. For the non-POPs, the range between minimum and maximum levels was in 1–2 orders of magnitude. Furthermore, they were also found in meconium samples (Figure 1).

**Table 2:** Endocrine disruptor levels in specimens (mg/L).

		Percent		Percentiles					
Sample	Analyte	>LOD	Minimum	5th	25th	50th	75th	95th	Maximum
Maternal blood (N = 201)									
	MEHP	65.66	0.17	0.95	1.32	1.82	2.87	4.16	6.74
	OP	64.14	0.10	0.13	0.28	0.47	0.66	1.37	2.97
	4-NP	65.66	0.06	0.14	0.24	0.38	0.64	1.63	5.58
Cord blood (N = 201)									
	MEHP	76.26	0.01	0.60	1.08	1.58	2.55	3.99	4.92
	OP	73.74	0.04	0.08	0.21	0.40	0.56	0.84	1.25
	4-NP	76.26	0.02	0.10	0.16	0.28	0.53	0.91	1.28

The sum of the 19 congeners (Σ_19_PBDEs) in serum ranged from 6.7 to 292 ng/g lipid for maternal blood and 6.9 to 581 ng/g lipid for cord blood. All PBDEs congeners were detected in serum except for BDE-138, 183, and 190, as shown in Table 3. And BDE-99, 153, and 154 were detected in only one sample. Among all targets, BDE-209 was the most abundant congener followed by BDE-207, 208, and 66, with the detective rate of 50%, 83%, 74%, and 74%, respectively. In all samples, the mean concentration of BDE-209 accounted for 38% of the total, which followed by BDE-207 (15%), 208 (11%), and 66 (9%).

**Table 3:** PBDE levels in maternal blood and cord blood specimens (ng/g lipid).

	Maternal blood (N = 29)		Cord blood (N = 29)		p value
	GM	95% CI	GM	95% CI	
**BDE-17**	0.47	0.07–2.80	0.35	0.81–3.40	0.151
**BDE-28**	1.78	1.86–8.66	1.31	4.83–13.89	0.061
**BDE-47**	0.75	0.59–3.22	0.62	1.81–7.04	0.119
**BDE-49**	0.94	0.92–2.92	0.16	0.25–1.70	0.254
**BDE-66**	2.57	3.11–7.54	1.26	3.66–11.35	0.313
**BDE-206**	0.98	1.15–8.49	0.52	1.62–10.86	0.309
**BDE-207**	3.32	4.35–13.21	3.13	6.30–14.77	0.576
**BDE-208**	2.17	2.82–11.16	1.94	3.70–9.55	0.457
**BDE-209**	2.12	4.84–26.25	1.33	5.23–75.94	0.202
**ΣBDE17-154**	10.37	8.55–24.35	11.92	14.10–34.64	0.253
**ΣBDE183-209**	9.43	14.59–60.51	13.87	20.75–108.16	0.111
**ΣPBDEs**	29.26	25.38–82.30	41.12	42.40–135.21	0.041*

GM = geometric mean.

* p<0.05.

**Figure 2:**
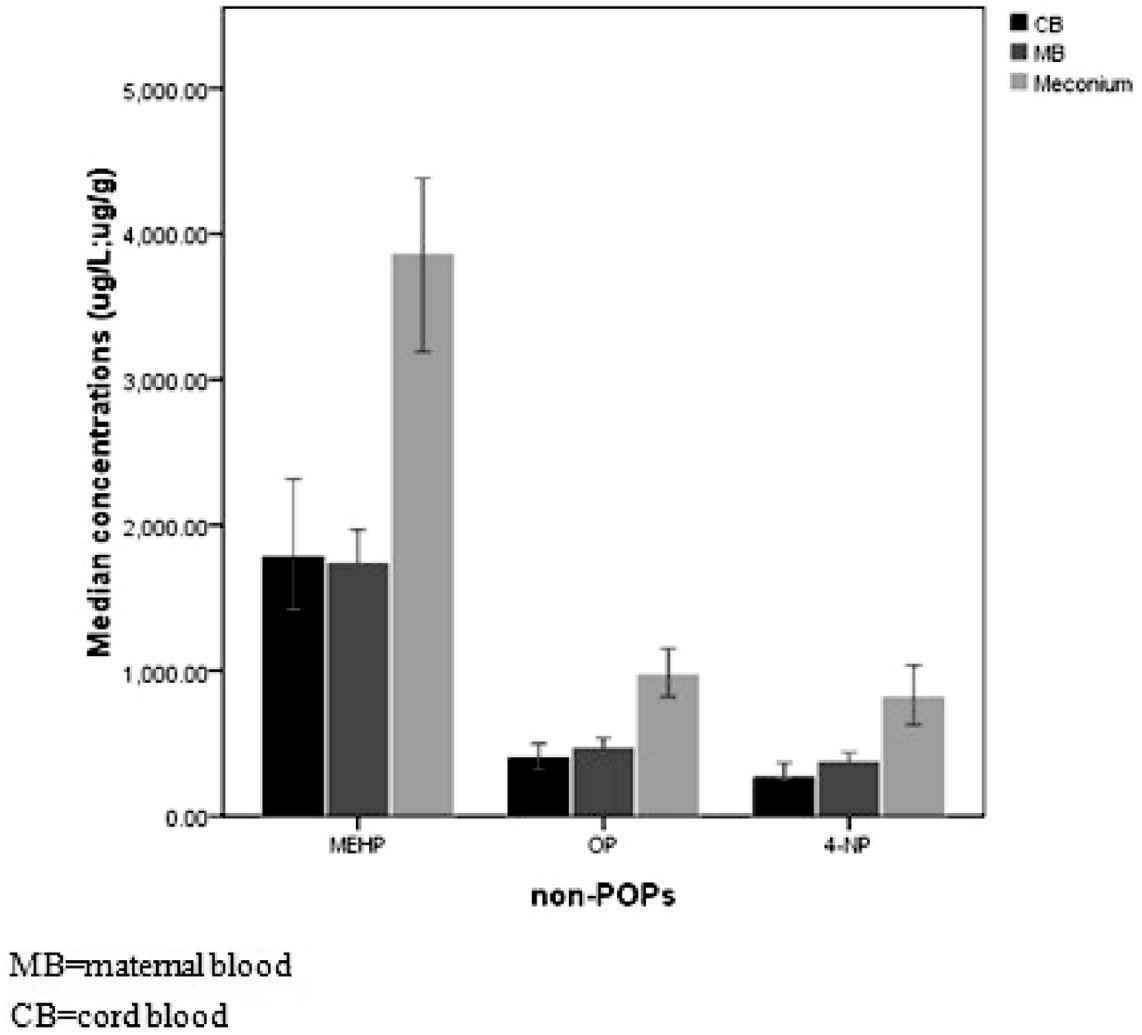
Non-POPs levels in all specimens. The concentrations of MEHP, OP and 4-NP in maternal blood, cord blood and meconium specimens. And their levels in maternal blood were significantly higher than those in cord blood (p<0.0001).

**Figure 3:**
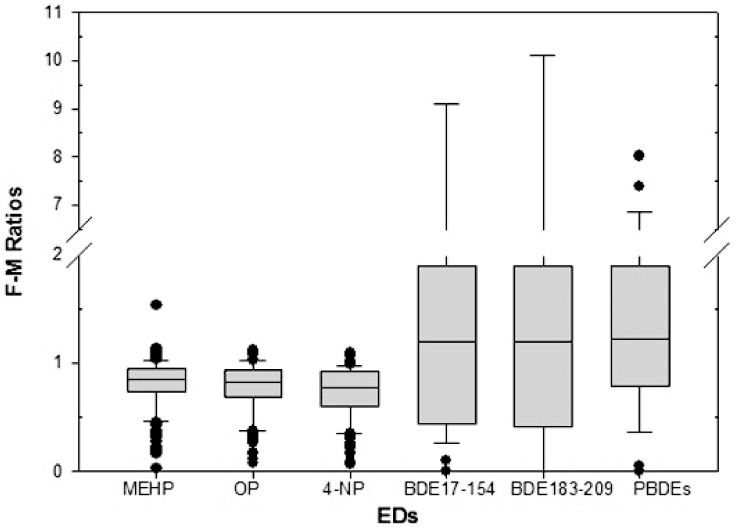
Fetal-maternal ratios of EDs. The ratio of MEHP, OP, 4-NP BDE17-154, BDE183-209 and ∑PBDEs levels in the fetal and maternal circulation, showing that their levels were approximately 20% lower in cord blood samples than those in maternal blood samples.

The concentrations of all these non-POPs in maternal blood samples were also significantly higher than those in cord blood (p<0.0001) (Figure 2), the ratio of which in the fetal and maternal circulation (F-M ratio) were calculated to evaluate the extent and kinetics of the transfer. The average placental transfer ratio between fetal and maternal blood for these EDs were presented in Figure 3, showing that their levels were approximately 20% lower in cord blood samples than those in maternal blood samples.

**Figure 4:**
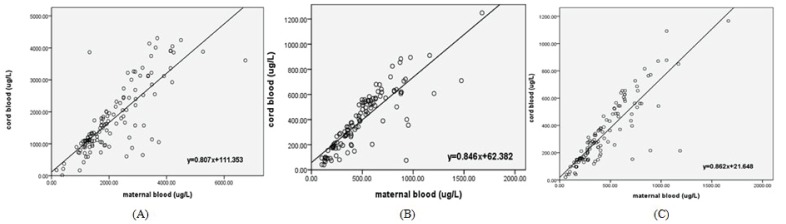
Regression analysis between non-POPs levels in maternal and cord blood. Regression analysis was used to analyze the relationship between these EDs levels in maternal and cord blood. (A) The relationship between MEHP levels in maternal and cord blood. The regression coefficient was 0.807, meaning that about 80% MEHP could cross the placenta; (B) The relationship between OP levels in maternal and cord blood. The regression coefficient was 0.846, meaning that about 85% OP could go through the placenta; (C) The relationship between 4-NP levels in maternal and cord blood. The regression coefficient was 0.862, meaning that more than 86% 4-NP could cross the placenta.

After controlling for the potential confounders (including gestational age, smoking, socioeconomic level, and prepregnancy BMI), regression analysis was used to analyze the relationship between these EDs levels in maternal and cord blood, and good correlations between the two specimens could be demonstrated by regression coefficients (Figure 4). The regression coefficients of all the non-POPs were over 0.80, indicating these EDs can cross the placenta to a great extent.

There was no apparent difference between the congener pattern of PBDEs in maternal and cord blood specimens, however, ΣBDE17-154, ΣBDE183-209 and Σ_19_PBDEs levels were significantly higher in cord blood than in maternal blood, with an F-M ratio of 1.20, 1.49, 1.65, respectively. And no statistic correlation was found between maternal and cord blood Σ_19_PBDEs levels.

**Figure 5:**
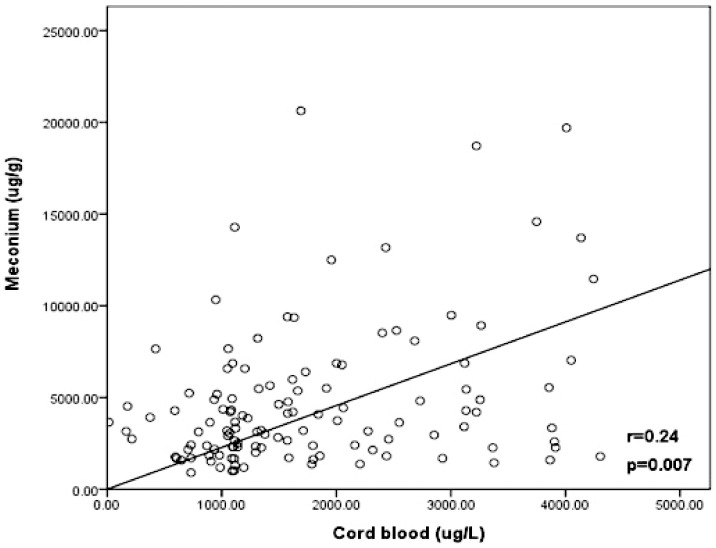
Correlation between MEHP levels in cord blood and in meconium. MEHP was detected in 135 of 201 mother-newborn pairs, presenting a good correlation in cord blood and meconium.

**Figure 6:**
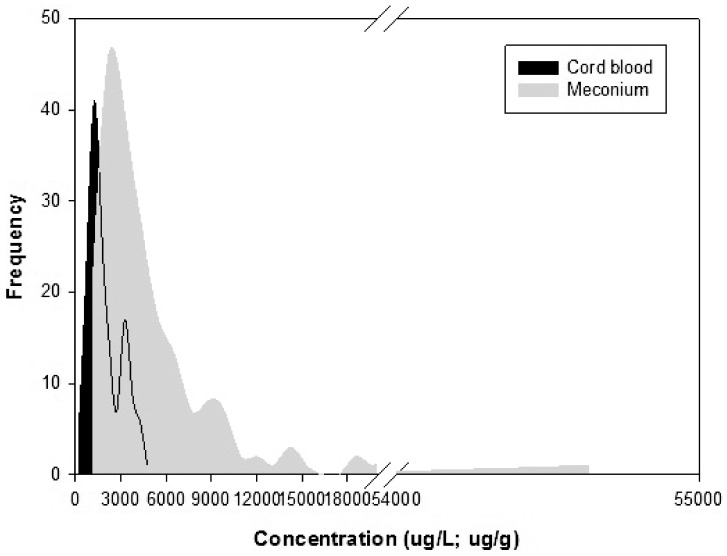
Frequency distribution of MEHP levels in cord blood and meconium. The frequency distribution of MEHP levels in cord blood and meconium, illustrating that MEHP levels in meconium were much higher than those in cord blood.

MEHP was detected in 135 of 201 mother-newborn pairs, presenting a good correlation in cord blood and meconium (Figure 5). And figure 6 showed the frequency distribution of MEHP levels in cord blood and meconium, illustrating that MEHP levels in meconium were much higher than those in cord blood.

## Discussion

Information on non-POPs and POPs in serum specimens from mother-newborn pairs in China is scarce [14]. We enrolled 230 mother-newborn pairs from Shanghai and Wenzhou during 2010–2011, measuring the non-POPs (including MEHP, OP and 4-NP) and POPs in their serum and meconium specimens. MEHP, OP and 4-NP were found in more than 65% maternal and cord blood samples in our study, indicating widespread human exposure to these non-POPs in Shanghai and confirming that human exposure to these chemicals can begin *in utero*. The median concentrations of non-POPs ranged from 0.38–1.82 mg/L in blood samples, which were one to two orders of magnitude higher than the results reported for Asia [3], [14] as well as for some European countries [15]–[17].

The median concentration of PBDEs (mean: 78.5 ng/g lipid) in our study was two orders of magnitude higher than those reported in serum samples collected from Denmark [18], Spain [19], Sweden [20], the Netherland [21] and France [22]. Compared with results for the general population of Korea [23] and USA [24], our results were comparable. BDE-209, 207, 206, and 66 were the dominant PBDEs congeners in this study, which wasn’t in accordance with the results of other reports. One possible reason for this phenomenon might be associated with the usage patterns of PBDEs commercial products in different areas. In China, deca-BDE was still the dominant brominated flame retardants (BFRs) and BDE-206, 207, and 208 were both from commercial octa- and deca-BDE products [25]. We found that BDE-209 was the most abundant congener in both maternal and cord blood, suggesting that industrial production of BDE-209 may result in the exposure of local residents to BDE-209.

Multiple linear regression was used to estimate the correlation between the concentrations of MEHP, OP and 4-NP in maternal and cord blood. A significant correlation was found between the concentrations of the non-POPs in maternal and cord blood, demonstrating maternal exposure during pregnancy and transplacental transfer may be an important route of exposure to fetus. Placenta plays a key role in material exchange between fetuses and mothers. Concentrations of the non-POPs in cord blood were significantly lower than those found in the maternal blood (p<0.0001). Furthermore, we used F-M ratio and regression coefficient to assess the potential function of placenta on barricading the mother-fetal transfer of these non-POPs. The nearer regression coefficient approached 1.00, the better they correlated, meaning that the more readily the chemical could get access to the fetus through the placenta. The average placental transfer ratios between fetal and maternal blood for these non-POPs were greater than 0.80, and their regression coefficients were all over 0.80, indicating that the placental barrier slightly decreased the fetal exposure to these non-POPs instead of totally preventing them.

However, it is apparent that PBDEs at environmental exposure levels could be transferred across the human placenta and reach the fetus, since F-M ratio of ΣBDE17-154, ΣBDE183-209 and Σ_19_PBDEs were all over 1.00. Similar to the results of former human epidemiology studies [26], [27], we found that PBDEs levels in cord blood were higher than those found in maternal blood, showing that the placenta did not pose an effective barrier to transplacental transport of this kind of POPs. The fact that statistically significant difference (p = 0.041) for Σ_19_PBDEs concentration between maternal and cord blood, together with the fact that no significant correlation was found in this study, might indicate that different BDE congeners could pass through the placenta at different penetration rates. These differences might be related to lipid content of samples, the liposolubility of each congener, and each PBDEs congener’s capacity for bioaccumulation.

Previous studies have already concluded that once a pregnant woman is exposed, phthalates, OP and 4-NP can cross the placenta and enter fetal circulation [3], [28], but little data came from population studies. Compared with them, we provided the concentrations of these chemicals in human’s serum specimens from 230 mother-newborn pairs, and used both the F-M ratios and regression coefficients to evaluate the extent of the transfer. The high F-M ratios and regression coefficients of these non-POPs demonstrated that they transported across the placenta to a greater extent, which clarified limited effect of placenta as a barrier on protecting fetus from *in utero* exposure to these commonly-used EDs.

Higher PBDE concentrations in the cord blood were also found in several previous studies [5], [29]. In the last trimester of pregnancy there is substantial redistribution of lipids from mother to the fetus [30]. Indeed the last trimester of pregnancy represents a catabolic phase within the maternal system which may redistribute contaminants from maternal stores to the fetus with the subsequent result that the concentrations of PBDEs in maternal blood are lower at delivery and substantially lower than those in the cord blood. We further postulate that if maternal exposure to PBDEs is high there could be important redistribution of PBDEs to the fetus. Alternatively, differences in the concentrations of PBDEs in maternal and cord blood may represent the inability of the fetus to metabolize these contaminants and adequately clear them from the circulation.

Since POPs are chemical substances that persist in the environment and bioaccumulate through the food web, fetuses could encounter high PBDEs exposure level through the repeated exposure from expectant mothers’ dietary intake, which also due to transplacental absorption, partitioning between the maternal and fetal compartments, as well as poor detoxification mechanisms of the developing organism. This could partly explain the difference between the non-POPs and POPs in placental transfer characteristics.

MEHP, and OP, 4-NP were also found in meconium samples in our study, which means the limit of the ability of the placenta to act as a barrier to these chemicals and indicates that they had already reached the developing fetus and been absorbed. In conclusion, placenta may not be a reliable barrier to these EDs, and transport of PBDEs across the placenta was much higher, which means PBDEs may do more harm to fetus and much easier to reach them than the other EDs.

As stated in previous reports about human DEHP metabolism, the simple monoester MEHP was the dominating metabolite in blood serum [31] as well as in cord blood serum samples [14]. We found MEHP in meconium samples, confirming that DEHP, like other phthalates, could cross the placenta into the fetal blood circulation and finally metabolized into their meconium as MEHP. Meconium, identified as a non-invasive matrix, is easier to collect and can give the information regarding long-term exposure. A fetus can be exposed to different chemicals, most of which are deposited and accumulated in the meconium. This process occurs through bile secretion and/or fetal swallowing of amniotic fluid, starting from the 12th week of gestation. A good correlation was also found between the MEHP levels in cord blood and in meconium, and its levels in meconium were much higher (p<0.0001), therefore, meconium can provide a longer and cumulative record of exposure to various environmental chemicals than cord blood. In one word, the significantly greater concentration of MEHP in meconium supports the use of MEHP level in meconium as an exposure biomarker for DEHP *in utero* exposure assessment.

The present study still has some limitations. We only collected 29 mother-newborn pairs in Wenzhou to detect PBDEs in their blood specimens. Such small number of samples may not convictive enough to draw the conclusion, so further research on PBDEs *in utero* exposure is warranted. The median concentrations of the MEHP, OP, 4-NP and PBDEs in our study were much higher than the results of previous studies. Phthalates are widely used in commercial products, as plastic softeners and solvents in personal care products, lubricants and insect repellents [32]–[34]. Potential sources of exposure for DEHP include polyvinylchloride containing medical devices, food packaging, plastic toys, furniture, and car upholstery. Recent studies suggested that the intensive use of plastic material in China may be increasing the exposure of DEHP in Chinese [35]. The disparity in PBDEs concentrations among different countries might be attributed to the different amount of PBDEs usage in different areas (USA 34,400 metric tons in 1999; China 10,000 tons in 2000; European Union banned since 2003) [36], [37]. However, concentrations of PBDEs in our study were in the middle range of similar studies which had been previously conducted in China [38], [39]. The higher levels of these EDs probably due to the higher environmental background value and higher pollution level in China [40].

Conclusively, mothers and newborns are widely exposed to MEHP, OP, 4-NP and PBDEs in China. Our study identified the placental barrier could slightly decrease the fetal exposure to most maternally exposed non-POPs, while to PBDEs, a major kind of POPs, its function is very limited. MEHP level in meconium represents an excellent and a robust parameter for assessing DEHP *in utero* exposure. Some EDs and/or markers of internal EDs exposure are presented in maternal and fetal blood. It is essential to study the transplacental kinetics of these compounds because information on the distribution among the fetal, the maternal, and the placental compartments is necessary to assess fetal risks associated with a maternal exposure to EDs. Therefore, further investigations on transplacental transfer of EDs are still needed.
